# Nuclear phase-out: Can we catch up on CO2 emissions?

**DOI:** 10.1371/journal.pone.0336218

**Published:** 2025-11-10

**Authors:** Luisa Loiacono, Leonzio Rizzo, Riccardo Secomandi

**Affiliations:** 1 Department of Economics and Management, Università degli Studi di Ferrara, Ferrara, Italy; 2 London School of Economics Visiting Fellow, London, United Kingdom; 3 Institut d’Economia de Barcelona, Universitat de Barcelona, Barcelona, Spain; Pukyong National University, KOREA, REPUBLIC OF

## Abstract

Nuclear energy is classified as a low-carbon energy source by institutions such as the European Union, due to its negligible direct carbon dioxide (CO2) emissions. Nevertheless, following major nuclear incidents such as Fukushima, several countries have implemented nuclear phase-out programs driven by safety concerns. This has raised critical questions about whether the reduction in nuclear capacity would be compensated for by an increase in renewable energy or by a return to fossil fuel-based generation. In this paper, we analyze the trajectory of CO2 emissions from fossil fuel energy sources (specifically coal, oil, and gas) in Germany after the launch of its nuclear phase-out in 2010. Using a synthetic control method, we find that although emissions from fossil sources declined overall in the country, the reduction was weaker in Germany after the phase-out, due to an increased reliance on fossil fuels, particularly coal. However, over a thirteen-year period, the gap in fossil-related CO2 emissions between Germany and its synthetic control group progressively narrows until 2023, when it disappeared. These findings suggest that the nuclear phase-out contributed to higher pollution from fossil fuel energy in the short term, but we find that this effect was gradually mitigated by a stronger growth of renewables in Germany during the 2011–2023 period.

## Introduction

Following the 2011 Fukushima nuclear disaster, Germany’s Chancellor Angela Merkel announced a decisive shift in the country’s energy policy, initiating a comprehensive nuclear phase-out. This policy, known as the “Atomausstieg,” received broad support across the political spectrum and was formalized by the Bundestag on June 30, 2011. The plan entailed the immediate shutdown of eight nuclear reactors and a scheduled decommissioning of the remaining nine by the end of 2022, aiming to eliminate nuclear power from Germany’s energy mix. The rationale behind this policy was rooted in public concern over nuclear safety and a longstanding anti-nuclear sentiment within the country. The phase-out was integrated into Germany’s broader “Energiewende” strategy, which seeks to transition the nation towards a sustainable energy system. This strategy includes ambitious targets: reducing greenhouse gas emissions by 80–95% by 2050 compared to 1990 levels and increasing the share of renewable energy in gross electricity consumption to at least 80% by the same year.

To compensate for the loss of nuclear-generated electricity, Germany planned to expand its renewable energy capacity, particularly wind and solar power, and improve energy efficiency. However, the transition also necessitated interim reliance on fossil fuels, notably natural gas and coal, to ensure energy security and grid stability. This reliance raised concerns about meeting emission reduction targets, as the increased use of fossil fuels could offset the environmental benefits gained from eliminating nuclear power. Germany successfully shut down its last three nuclear power plants (Isar 2, Emsland, and Neckarwestheim 2) on April 15, 2023, marking the completion of the nuclear phase-out.

Nuclear power is associated with very low operational carbon dioxide equivalent emissions per kWh, around 12, much lower than coal (820), gas (490), or even solar (48) and hydropower (24), according to United Nations Intergovernmental Panel on Climate Change [1]. Therefore, when nuclear plants are phased out and the produced electricity is initially replaced by the available coal or gas power plants, it is very likely to experiment an increase in CO₂ emissions in the short and/or medium-long term.

Energy system transformations, such as the unexpected and immediate German nuclear power phase-out, force reliance on existing, high-carbon energy sources because the system lacks alternative low-carbon generation capacity, thus generating unintended environmental costs.

The rigidity and technological inertia of energy systems facing energy transition have been extensively discussed in the literature. Unruh [2] conceptualizes this phenomenon as carbon lock-in, a persistent condition in which industrial economies become locked into fossil fuel-based technological systems. To explain this phenomenon, the author introduces the Techno-Institutional Complex, which captures the idea that lock-in occurs through the combined interaction of technological systems and governing institutions that are difficult to displace and can lock out alternative technologies for extended periods. The question of how to overcome this systemic inertia is addressed by Unruh [3]. Exploring the policy implications of the lock-in concept, Unruh [3] argues that the conditions necessary to escape the lock-in state are unlikely to be generated internally. Instead, exogenous forces are required to overcome institutional resistance to radical change. Historically, institutional theory suggests that large-scale reorientations often require major crises or external shocks, before decisive policy actions are taken. The German acceleration of the nuclear phase-out immediately following the Fukushima disaster represents just such a political response to an exogenous shock. Building on this, more recent empirical work investigates how systemic vulnerabilities may intensify the negative effects of carbon lock-in. Zhao et al. [4], by using a provincial panel dataset in China over the period 2002–2017, identify the causal relationship between carbon lock-in and energy security (the system’s ability to respond to external shocks, like a sudden black-out), energy equity proxied by the level of accessibly and energy environmental sustainability. They find that the more the electricity provision is triggered by potential exogenous shocks, the more the price each customer must pay is territorially differentiated and the more visible is the negative environmental impact, the higher is the probability of a crisis of the system backing the barrier to carbon unlocking.

The nuclear phase-out policies have been closely analyzed in academic literature, with studies examining their impact on emissions and energy mix. Kunz and Weigt [5] look at the consequences of Germany’s nuclear phase-out policy. Their analysis combines descriptive ex-post evaluation using data from 2009 to 2012 with an assessment of the future trajectory of the phase out. They find that the immediate closures in 2011 led to a reduction in nuclear generation, compensated mainly by fossil-fueled power plants and reduced net exports. CO_2_ emissions rose in the short term due to the increased use of fossil fuels. In their simulations of the full phase-out, they project that generation adequacy will not be at risk, but they highlight concerns about increasing redispatch needs, driven by grid congestion, especially in southern Germany, due to the geographic mismatch between retiring nuclear plants and expanding renewables. According to them it is therefore important to take care of the transmission grid extensions and market design improvements to maintain system stability. Qvist and Brook [6] analyze Sweden’s rapid nuclear expansion between 1960 and 1990 as a case study in deep decarbonization of the power sector. Using historical data and a descriptive comparative approach, they show that the transition from fossil fuels to nuclear power led to a reduction in CO₂ emissions of more than 75% in less than two decades, while maintaining stable electricity prices and reliability of supply. The authors argue that this transformation was enabled by the dispatchable and high-capacity nature of nuclear energy, which allowed for the phase-out of fossil fuels power plants, unlike intermittent renewables sources. They suggest that the Swedish experience illustrates the feasibility of large-scale nuclear plants as a strategy for decarbonization. The French experience is also mentioned as comparable, with France achieving similar per capita reductions in CO₂ emissions from electricity production through a similar expansion of nuclear power. Davis and Hausman [7] study the closure of the San Onofre Nuclear Generating Station (SONGS), the second-largest electric generating facility in California, which occurred in February 2012 due to problems with the plant’s steam generators. The authors use a regression-based model of electricity supply and demand, estimated using monthly data from 2002 to 2014, and analyze changes in generation, emissions, and costs. Their main finding is that CO_2_ emissions increased by an average of 1,030 metric tons per hour during the twelve months following the closure. Summing across all hours and plants, the study concludes that carbon dioxide emissions increased by approximately 9 million metric tons during the first year. The additional generation came largely from in-state natural gas plants. The external cost of these emissions is valued at $316 million, based on a social cost of carbon of $35 per ton. Lee et al. [8] investigate the impact of nuclear power generation on CO_2_ emissions using data from 18 countries (OECD and non-OECD) over the period 1970–2015. The study employs panel cointegration analysis to determine whether a long-run equilibrium relationship exists between nuclear power generation and CO_2_ emission. The results show that nuclear power generation has a statistically significant negative effect on CO_2_ emissions in the long run, indicating that an increase of 1% in nuclear power leads to a 0.32% decrease in CO_2_ emissions per capita during the analysis period. Similarly, Marques and Junqueira [9] find that reducing nuclear capacity in the European Union leads to higher fossil fuel use and electricity prices. They use data from 2014 to 2018 and apply a decomposition model based on simulation to assess scenarios where nuclear energy is progressively phased out. Their analysis focuses on the EU and Switzerland. The results show that when nuclear is removed and replaced by fossil fuels, electricity-sector carbon intensity (and therefore CO_2_ emissions) increases due to the loss of a low-carbon baseload source. The authors highlight both environmental and economic consequences, including modest increases in electricity prices. Petruška et al. [10] investigate the impact of renewable and nuclear energy on CO_2_ emissions in 22 EU countries using panel data from 1992 to 2019. They apply panel cointegration and Granger causality tests to assess long-term effects. The results show a statistically significant negative long-run relationship between both nuclear and renewable energy consumption and CO_2_ emissions, suggesting that both energy sources contribute to reducing emissions in the EU. Kartal et al. [11] use French data from 1970 to 2021 and investigate the environmental impact of potential shifts in France’s energy mix under the constraint of Russian gas supply cuts. The study applies a Dynamic Simulations approach which simulates counterfactual scenarios, including reductions in natural gas consumption and increases in nuclear and coal consumption. The authors distinguish between short- and long-term effects, showing that increases in nuclear energy significantly reduce CO_2_ emissions in both horizons. These effects emerge strongly in the first two years and then level off, indicating a lasting but stabilized impact. In contrast, gas, coal, and oil increases lead to significant emission rises. Renewable energy displays a negative coefficient, but the effect is not statistically significant, suggesting that, within the observed period, variations in renewable energy consumption did not produce a consistent or robust impact on CO_2_ emissions.

The case of Germany has also been extensively analyzed, though primarily relying on simulation-based methodologies. Germany moved from early state-supported nuclear development in the 1950s to growing public opposition and the eventual decision to phase out nuclear power entirely by 2022. This policy shift was driven by the Fukushima accident in 2011 and framed within a broader transformation of energy governance [12]. Renn and Marshall [12] note that phasing out nuclear energy without having secured a sufficient supply of renewable energy may compromise climate protection goals as they show that carbon emissions may increase as fossil fuels, particularly coal, are used to compensate for the loss of nuclear power. Knopf et al. [13] analyze how different timelines for phasing out nuclear power in Germany affect CO_2_ emissions, the energy mix, and system costs. They use an energy-economy-climate model that calculates the most cost-effective way to meet energy demand over time. The model simulates scenarios where nuclear power is phased out in 2015, 2022 or extended until 2038 and it projects how the energy system could evolve under different nuclear phase-out strategies. The results show that ending nuclear power in 2015 instead of 2020 or 2022 would result in 64 million tons more CO_2_ emissions. If nuclear plants had remained in operation until 2038, between 45 and 70 million tons of emissions could have been avoided. The study finds that an earlier phase-out increases fossil fuel use in the short term, which raises both emissions and system costs. However, over the long term, as renewable energy expands and fossil-fuel-based generation declines, emissions across the different scenarios become similar. Jarvis et al. [14] also show that the shift in electricity generation resulted in an annual social cost of roughly €3–€8 billion per year, due to excess deaths caused by increased coal-related air pollution. Their estimations are based on event-study evidence and machine learning applied to data from 2011 to 2019. The phase-out also led to a roughly 11% increase in CO_2_ emissions mainly due to an increase in electricity produced from hard coal and oil power plants. Renuart and Li [15] analyze the impact of Germany’s 2011 nuclear shutdown using a synthetic control method with a European donor pool and data from 2000 to 2019. They construct a counterfactual scenario to estimate Germany’s air pollutant emissions in the absence of the phase-out. The results show that replacing nuclear power with fossil fuels slowed the reduction of several air pollutants, including NOₓ and CO₂. The effect is persistent and does not disappear over time, indicating a long-term increase in emissions relative to synthetic control.

Our work concentrates on the pollution directly linked to fossil fuels, isolating the impact on CO_2_ linked to fossil fuels which are those energy sources which substitute nuclear energy at first. As shown by previous literature, nuclear phase-out generally leads to increased fossil fuel usage and consequently higher CO₂ emissions. However, the literature focuses on the short-run effect of the nuclear phase-out; the only notable exception is the simulation by Knopf et al. [13], who find that, in the long run, emissions tend to converge across different scenarios. Our dataset, spanning from 1990 to 2023, reveals an interesting pattern in fossil fuel-related CO_2_ emissions following the nuclear phase-out. Initially, there is a six-year period during which Germany’s CO_2_ emissions from fossil fuels rise compared to the synthetic Germany scenario. However, from 2017 onward, the emission trajectories of both Germany and its synthetic counterpart begin to converge, even though the nuclear phase-out continues, with the final nuclear plant closing in 2023.The novelties of our approach are twofold. First, it is crucial, unlike in other studies such as Renuart and Li [15], to focus specifically on CO_2_ emissions arising directly from the fossil fuels used to replace the energy lost due to the nuclear phase-out. We specifically exclude emissions from industrial processes such as cement production and gas flaring. In contrast, Renuart and Li [15], who also used the Synthetic Control Method (SCM) to study Germany’s 2011 nuclear shutdown, utilized total CO_2_ emissions. Second, evaluating the short- and long-term effects is essential. In the short run, the phase-out generates a significant increase in pollution. However, in the German case this pollution increase dissipates in the long run (after approximately seven years), primarily due to the substantial expansion of renewable energy sources and the subsequent reduction in coal-based energy production. It is important to emphasize that the temporary social costs associated with increased emissions immediately following the nuclear phase-out could have been mitigated through a more carefully planned energy substitution strategy. Indeed, despite the ongoing closure of nuclear plants, particularly evident after 2020 (S1 Fig), our findings show that proper planning and management can prevent a rise in fossil fuel-related emissions.

While we focus on CO₂ emissions from fossil fuels and electricity generation, the nuclear phase-out can also affect the electricity price level and national electricity trade balance. It has to be noted that the effect of the nuclear phase-out on prices is not easy to identify since the strong increase of renewable generation and the inclusion of carbon costs in the electricity price could have influenced the price by decreasing it. Fürsch et al. [16], using a detailed optimization model for European electricity markets (DIME) analyze two scenarios (phase-out versus no phase-out), concluding that electricity prices would be higher in the phase-out scenario. Based on their analysis up to 2030, which incorporates a high share of renewable energy sources (RES), they forecast that the German wholesale price level would increase by 4–9 EUR/MWh in the phase-out relative to the baseline scenario. Traber and Kemfert [17], who apply a dynamic long-term Cournot-Nash equilibrium model of the European electricity sector to investigate effects up to 2020, find that the phase-out policy would induce modest wholesale price increases in Germany between four and twelve percent. However, empirical ex-post analyses show that the impact on prices is milder than previously forecasted by theoretical models (Kunz and Weigt [5]). These findings follow Nestle [18], who, after conducting an empirical analysis of EEX (European Energy Exchange) spot and future price data, shows that the availability of nuclear power plants does not affect electricity prices in Germany.

A second major concern is that the decommissioning of domestic nuclear generation would lead to increased reliance on energy imports, potentially undermining Germany’s energy security. Fürsch et al. [16] show that nuclear capacity replacement would lead to an increase in domestic fossil fuel-based generation and power imports, and a reduction in exports: German net imports would increase from approximately 10 TWh in 2015 to more than 20 TWh after 2020. Similarly, Traber and Kemfert [17] find that Germany would experience a negative physical trade balance (net importer) in all scenarios, regardless of nuclear policy, but the phase-out would still lead to a decline in the trade balance of about 7.5 TWh in the scenarios featuring successful energy efficiency policy.

## Methodology

We use the Synthetic Control Method (SCM), originally proposed by Abadie and Gardeazabal [19] and later developed by Abadie, Diamond, and Hainmueller [20], to estimate the effect of a policy intervention when only one unit is treated. SCM is especially useful when there is no obvious control group and only one treated case is available, such as in our case with Germany.

SCM creates a synthetic version of the treated unit, called the synthetic control, which serves as a counterfactual to estimate what would have happened in the absence of the intervention. The synthetic control is built as a weighted average of units in the donor pool, which in our case includes European countries not affected by the same policy but with nuclear energy production. The weights are chosen so that the synthetic control matches Germany as closely as possible in the pre-intervention period, both in terms of CO_2_ emissions and other relevant predictors.

The idea is that, if the synthetic control closely follows Germany before the intervention, then any difference between the two in the post-intervention period can be interpreted as the effect of the policy. This difference gives us an estimate of the causal effect of the intervention on CO_2_ emissions.

As explained in Abadie [21], the method relies on some key assumptions. First, the treated and synthetic units should have similar trends in the outcome variable before the policy. Second, the outcome should not be highly volatile over time. Third, a suitable donor pool must be available to construct a valid counterfactual. Finally, the countries in the donor pool must not be affected by the intervention. This last point is important: the countries used to build up the synthetic control must not experience any indirect effects of the treatment. This is known as the no spillover assumption, also referred to as SUTVA (Stable Unit Treatment Value Assumption). It means that the treatment in Germany should not influence emissions in the other countries used as controls.

Formally, following Abadie, Diamond, and Hainmueller [22], we consider a sample K + 1 units, indexed by k, where k = 1 is treated unit, and k = 2... K + 1 are the units belonging to the donor pool. The units are observed at the same time periods, t = 1... T0…T, with the pre-intervention period Tb when t < T0 and the post-intervention period Ta when t ≥ T0, where T0 is the time when the treatment starts. We define X1 = (s x 1) as the vector of the s variables (predictor variables) accounting for the pre-intervention characteristics of the treated unit, and X0 = (s x K) as the matrix collecting the values of the same s variables for all the other units in the donor pool. When more than one time-unit in the pretreatment period is available, the mean value of the s variable along the pretreatment time-units is taken. A vector of weights W is chosen to minimize the weighted mean square error (X1 - X0W)’ V (X1 - X0W), where V is a diagonal of predictor weights, which reflects the relative importance assigned to the predictor variables when the discrepancy between X1 and X0W is measured.

Following Abadie, Diamond, and Hainmueller [14], V is selected by solving


$$V=\underset{V}{\text{argmin}}{\left(Z_{\text{1}}-Z_{\text{0}}W(V)\right)}^{\prime }(Z_{\text{1}}-Z_{\text{0}}W(V)$$

where Z1 is a vector and Z0 is a matrix of the dependent variable before the treatment for the treated and for the donor group, respectively. The idea is to find values of *V* that minimize the mean squared prediction error (MSPE) between the treated unit and its synthetic counterpart in the pre-intervention period. This means predictor weights V are chosen endogenously from the data, so that the weighted combination of donor units best reproduces the pre-treatment trajectory of the treated unit.

## Data

To apply the Synthetic Control Method (SCM), we use a panel dataset covering Germany and a group of European countries that were not affected by the same intervention but had positive nuclear energy production during the period considered. We exclude countries that experienced a nuclear phase-out (Belgium, phase-out law 2003; Lithuania, final shutdown 2009) and EU-27 members with zero nuclear electricity production over 2000–2009. This leaves 12 countries in our dataset (including Germany). The countries included in the donor pool are Bulgaria, Czechia, Finland, France, Hungary, Netherlands, Slovak Republic, Slovenia, Spain, Sweden, and Romania. The time span covers the years from 1990 to 2023, with the policy intervention in Germany set in 2011.

The outcome variable is annual carbon dioxide emissions from fossil fuel sources that cover energy-related CO₂ emissions, calculated as the sum of emissions from natural gas, oil, and coal (for descriptive statistics see S1 Table). We retrieve the data from Our World in Data [23]. We exclude CO_2_ emissions from industrial processes such as cement production and gas flaring. In our empirical setting, we do not posit any disruptive, contemporaneous shifts outside the power sector that would confound identification; rather, fuel use in transport, buildings, and industry evolves gradually over our sample window. Indeed, we also use as alternative outcomes the electricity generated from different energy sources, such as gas, coal, oil, renewable energy sources, and nuclear power as we aim to examine the direct correspondence between CO_2_ emissions and electricity generated, since changes in emissions might also be influenced by technological improvements and other factors. Germany is the treated unit, and the remaining countries serve as potential donors for the construction of the synthetic control.

To ensure that the synthetic control closely replicates the pre-intervention path of Germany, we include a set of socio-economic and energy-related variables as predictors. These include gross domestic product, number of registered cars, total electricity consumption, electricity production from nuclear sources, electricity production from fossil fuel sources, energy intensity, average temperature, industrial value added, population density, percentage of population living in urban areas, and percentage of electricity consumption by households. The data are drawn from Eurostat, the OECD, and national statistics sources. For the pre-treatment period, we use average values for the years 1990, 1995, 2000, 2005 and 2009.

Following Abadie, Diamond, and Hainmueller [22], we also include values of the dependent variable from 1990, 1994, 1998, 2002, 2006, 2010 to improve the accuracy of the synthetic control. Once the weights are estimated, they are applied to the donor countries to generate the synthetic emissions trajectory for Germany over the full period of analysis. The effect of the policy is then measured as the difference between actual emissions in Germany and the emissions of the synthetic control after 2011.

## Results

Using the total CO_2_ emissions from fossil fuel sources as the outcome variable, the synthetic Germany is built using a weighted average of Czechia (51.7 percent), Netherlands (31.2 percent), France (10.9 percent), Sweden (4.8 percent), and Spain (1.4 percent). The remaining countries in the donor pool (Bulgaria, Finland, Hungary, Slovak Republic, Slovenia, and Romania) receive zero weight. The differences in the mean values of each predictor between Germany and the synthetic Germany are small (Table 1), confirming the validity of the SCM procedure and the reliability of the constructed counterfactual. The only relevant discrepancy arises in energy intensity, which is attributable to the composition of the donor pool, notably Czechia and the Netherlands, both of which exhibit structurally high energy intensity [24,25], in contrast to Germany, which has shown strong improvements in energy efficiency over time [26].

**Table 1:** Predictors (Germany and synthetic Germany) for per capita carbon dioxide emissions from fossil fuel sources.

Predictors	Germany	Synthetic Germany
GDP (per capita)	28524.79	23860.41
Registered cars (per capita)	0.69	0.67
Total electricity consumption (per capita)	7071.88	7089.99
Electricity consumption from fossil fuels (per capita)	3864.88	3331.70
Electricity consumption from nuclear (per capita)	1944.38	1636.70
Average temperature	9.15	9.02
CO2 emissions from fossil fuel (1990)	12.86	12.32
CO2 emissions from fossil fuel (1994)	11.18	11.00
CO2 emissions from fossil fuel (1998)	10.91	10.71
CO2 emissions from fossil fuel (2002)	10.65	10.65
CO2 emissions from fossil fuel (2006)	10.54	10.59
CO2 emissions from fossil fuel (2010)	9.94	10.02
Share of industrial value added	27.60	28.67
Population density	233.40	232.18
Share of population in urban areas	73.28	75.61
Energy intensity	3.80	5.09
Share of electricity consumption by households	0.26	0.24

**Fig 1:**
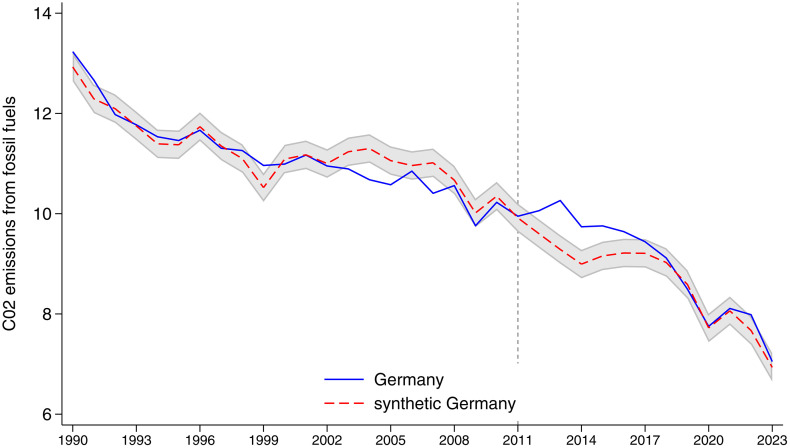
Germany vs synthetic Germany, per capita carbon dioxide emissions from fossil fuel sources. Notes: The blue line represents the per capita carbon dioxide emissions from fossil fuel sources of Germany, while the red line represents the per capita carbon dioxide emissions from fossil fuel sources of the synthetic Germany. The shaded area [27] is built by adding and subtracting to the outcome of the synthetic Germany one standard deviation of the difference between the outcome of Germany and synthetic Germany before the introduction of the policy.

Graphically, before the policy intervention in 2011, the CO_2_ emissions from fossil fuels in Germany and in synthetic Germany follow a very similar trend, with only minor differences, which are in the opposite direction compared to the results observed in the post-treatment period. After 2011, however, actual emissions in Germany decline less sharply than in the synthetic control, indicating an effect of the policy (Fig 1). The shaded area is constructed by adding and subtracting one standard deviation of the difference between Germany and synthetic Germany in the pre-treatment period to the value of the synthetic Germany. This interval represents the range within which Germany would be expected to fall on average before the intervention, assuming no treatment effect [27].

Between 1990 and 2010, emissions (tons) in both Germany and the synthetic control gradually decreased, remaining close to each other. After 2011, we observe a divergence marked by a two-year increase in actual emissions, which is not mirrored in the synthetic control. From 2013 onward, Germany’s fossil fuel emissions begin to decline again, although they remain significantly above the synthetic counterfactual. This gap persists until 2017. Notably, starting in 2017, the difference between the two trajectories narrows considerably, with the two lines nearly overlapping in 2021. In the final two years of observation, 2022 and 2023, a slight divergence re-emerges, likely linked to the completion of the nuclear phase-out. However, this divergence, where Germany’s emissions are slightly higher than those of the synthetic control, remains within the one-standard-deviation confidence band.

This convergence suggests that the initial effect of the policy faded over time. A plausible explanation is that, following the initial phase-out of nuclear power, Germany progressively compensated for the lost capacity by expanding renewable energy sources. As a result, despite the continued decommissioning of nuclear plants, actual emissions no longer diverged from the counterfactual. Eventually, Germany’s emissions trajectory realigned with that of comparable countries in the donor pool, eliminating the long-term differential effect. This interpretation is confirmed by the evolution of electricity production by energy source. Our results are confirmed by applying the Synthetic Difference-in-Differences (SDID) method, proposed by Arkhangelsky et al. [28]. The SDID extends standard difference in differences by assigning weights to each control unit and time unit so that the weighted control outcomes are approximately parallel to the treated unit. Considering the entire period available after the introduction of the policy (2011–2023), we observe an average null effect both on carbon dioxide emissions (S2 Table, Col. 1) and on its decomposition into gas (Col. 3), coal (Col. 5), and oil (Col. 7). However, we do confirm the increase shown by the synthetic control method in the immediate post-policy years (2011–2017). Using per-capita total CO2 emissions as the dependent variable (Col. 2), the coefficient on the interaction between the treatment indicator (Germany) and the treatment window (2011–2017) is positive (0.514) and statistically significant at the 5% level. Moreover, when decomposing fossil-fuel emissions, only for per-capita CO₂ emissions from oil the coefficient of the interaction between treatment and post (0.286) is positive and statistically significant at 5% (Col. 8). This pattern indicates that the increase in CO₂ emissions is mainly due to greater reliance on oil. By contrast, the coal uptick is not detected by the SDID, as the coal peak was confined to 2011–2014.

**Fig 2:**
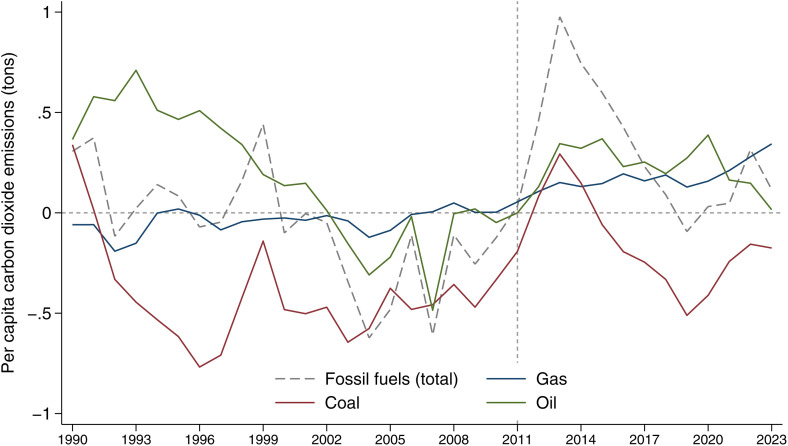
Difference between Germany and synthetic Germany, per capita carbon dioxide emissions from fossil fuel sources and its composition: gas, coal, and oil. Note: See S2 Fig for individual sources synthetic control (coal, gas, oil).

Fig 2 provides a decomposition of the CO_2_ emission gap between Germany and its synthetic control into its main fossil fuel components: coal, gas, and oil. In the ten years preceding the policy intervention (2011), Germany consistently exhibited lower per capita CO_2_ emissions from fossil fuels compared to the synthetic control. This pattern holds for total fossil fuel emissions as well as for each individual source (coal, gas, and oil) starting around the year 2000. After 2011, the difference turns positive, indicating that emissions in Germany increased relative to the synthetic control. The breakdown reveals that this divergence is primarily driven by a sharp rise in coal-related emissions following the nuclear phase-out. Coal emissions peak around 2013–2014, then decline significantly by 2016, before rising again in 2019, likely reflecting the closure of additional nuclear plants. Oil-related emissions, by contrast, display a generally declining trend, except for a small spike around 2020. Gas emissions show a different pattern: they remained below the synthetic control until 2011, then started to rise moderately and remained above the counterfactual. This suggests that gas partly substituted for both the declining use of coal and the gradual nuclear phase-out. Moreover, the increase in gas over time is likely not only a direct compensation for the nuclear capacity being phased out but also reflects the role of gas in ensuring energy security. As renewable energy sources are non-dispatchable and weather-dependent, maintaining grid stability requires flexible and dispatchable generation sources. Importantly, by 2023, the total per capita CO_2_ emissions from fossil fuels in Germany converge back toward the synthetic control level, with the gap nearly closing. This indicates that the initial increase in emissions was temporary and was later offset by changes in the energy mix, particularly the expansion of renewables.

**Fig 3:**
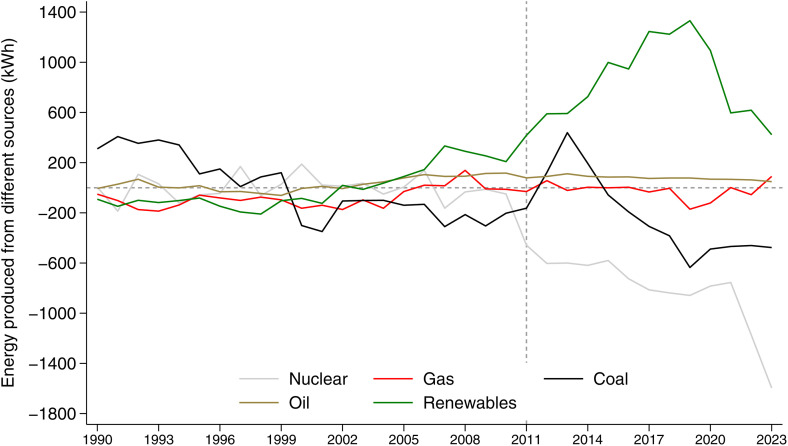
Difference between Germany and synthetic Germany, per capita electricity produced (kWh) from nuclear, gas, coal, oil and renewable energy sources. Note: See S3 Fig for individual enery sources synthetic control (nuclear, gas, coal, oil, renewable).

The emission dynamics described are mirrored in the energy mix. Indeed, changes in CO_2_ emissions closely correspond to shifts in the composition of electricity generation (Fig 3). By plotting the difference in electricity produced between Germany and its synthetic control by energy source, we observe no substantial pre-trend divergences—except for a lower use of coal in Germany from the early 2000s onward, and a modestly higher contribution of renewables starting in 2005. A sharp divergence emerges immediately after the policy intervention in 2011. From that point on, Germany exhibits a significant increase in electricity generated from renewables compared to the synthetic control. This upward trend continues until around 2019, after which the difference slightly declines but remains positive. As expected, nuclear power follows a clear downward trajectory, with a sharp drop in 2023 corresponding to the final phase of the nuclear phase-out and is compensated by the jump in the coal source. In fact, coal shows a marked increase in the years immediately following the policy, in line with the observed path in CO_2_. This increase is gradually reversed, and by 2023 coal use is substantially lower again. Gas remains relatively stable throughout the period, with only a slight increase from 2021 onward. This stability suggests that gas did not play a dominant role in replacing nuclear energy, although it did provide some support, especially in terms of system flexibility and grid stability. Interestingly, the result we find coincides with the forecasts of Knopf et al. [13].

### Robustness tests

We conduct a series of standard robustness checks. First, we want to ensure that the estimated effect is not also present in other countries in the donor pool. To test this, we replicate the synthetic control procedure using each donor country as if it were treated (placebo in space). Second, we aim to rule out the possibility that the observed effect is driven by events occurring before 2011. To do so, we run the synthetic control method using Germany as the treated unit but simulate the intervention at different points in time prior to 2011 (placebo in time). Finally, we perform a leave-one-out analysis, re-estimating the model while excluding one country at a time from the donor pool. This allows us to verify that our results are not driven by the inclusion of any specific country.

#### Placebo in space.

We first estimate the synthetic control for each of the countries in the donor pool, treating each of them as if they had received the intervention. This placebo analysis helps assess whether the effect observed for Germany is unusually large compared to the rest of the sample.

To do so, we compute the ratio between the post-treatment and pre-treatment root mean squared prediction error (RMSPE). A higher ratio indicates a greater discrepancy between the observed and synthetic values after the intervention relative to the fit before the intervention. Germany has a ratio of 3.415, which is the highest among all countries in the sample (Table 2). The second highest is Sweden, with a ratio of 1.747. All other countries display values below one. This indicates that the divergence between Germany and its synthetic control after 2011 is clearly more pronounced than for any other unit in the donor pool, confirming the reliability of our estimate.

**Table 2:** Placebo tests for per capita carbon dioxide emissions from fossil fuel sources.

Country	Ratio	RMSPE pretreatment	RMSPE post-treatment
Germany	3.415	0.218	0.744
Sweden	1.747	0.450	0.787
Finland	0.985	1.699	1.674
Romania	0.905	1.029	0.932
Hungary	0.835	0.411	0.343
Bulgaria	0.765	0.726	0.555
Czechia	0.671	1.942	1.302
France	0.611	0.312	0.191
Slovak Republic	0.566	0.756	0.428
Spain	0.405	0.873	0.354
Netherlands	0.357	1.372	0.489

The RMSPE is equal to the square root of the mean of the square of the difference between the treated and the synthetic control. It measures how closely the synthetic control replicates the treated unit. A low pre-treatment RMSPE indicates a good fit, while an increase in post-treatment RMSPE suggests a potential effect of the intervention.

#### Placebo in time.

**Fig 4:**
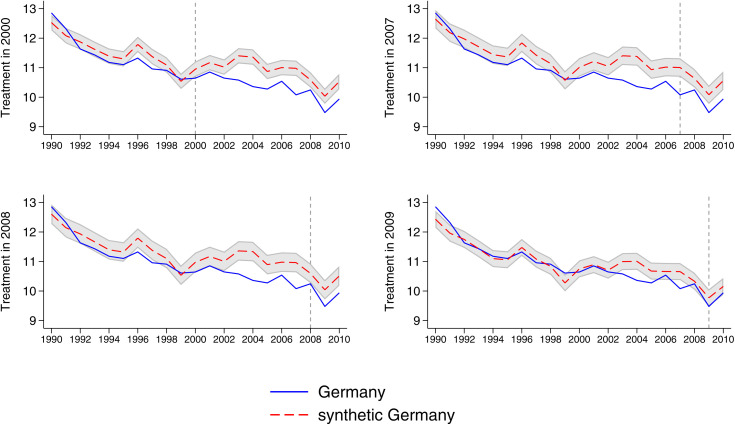
Germany vs synthetic Germany, per capita carbon dioxide emissions from fossil fuel sources, placebo in time: policy intervention estimated in 2000, 2007, 2008, 2009. Notes: The blue line represents the per capita carbon dioxide emissions from fossil fuel sources of Germany, while the the red line represents the per capita carbon dioxide emissions from fossil fuel sources of the synthetic Germany. The shaded area [27] is built by adding and subtracting to the outcome of the synthetic Germany one standard deviation of the difference between the outcome of Germany and synthetic Germany before the introduction of the policy.

We run four placebo tests in time (Fig 4). The spanning period in this case goes from 1990 to 2011 and the first placebo assumes a fake shock in 2000. The result shows that after 2000, CO_2_ emissions in Germany are lower than in Synthetic Germany, which goes exactly in the opposite direction of the result we obtain by looking at the phase out effect after 2010. We then perform placebo tests by assigning a fake treatment in 2007 and 2008. In both cases, Germany lies below the synthetic Germany, indicating no upward divergence prior to the real intervention. In a final placebo test, we simulate the start of the treatment in 2009 and find no meaningful difference between Germany and its synthetic counterpart.

#### Leave-one-out test.

**Fig 5:**
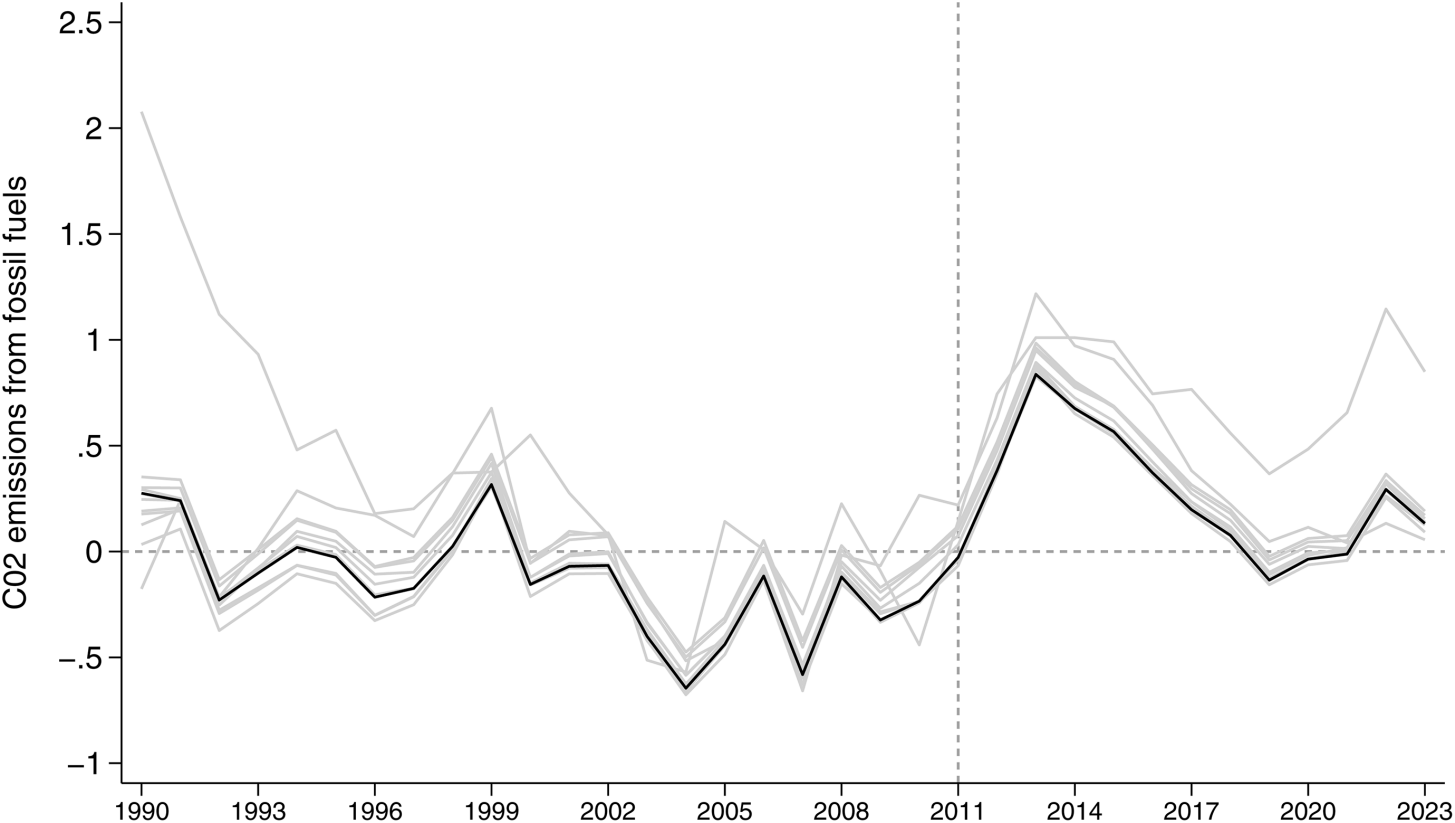
Difference between Germany and the synthetic Germany, excluding one country for each specification.

Finally, we perform a leave-one-out analysis, excluding each donor country from the pool one at a time (Fig 5). The results remain largely unchanged. However, it is worth noting that when the Czech Republic is excluded, the main findings still hold, but a noticeable difference between Germany and the synthetic control persists in 2021 in terms of CO_2_ emissions.

## Conclusions

Our results suggest that the 2011 nuclear phase-out in Germany initially led to an increase in CO_2_ emissions from fossil fuels compared to a synthetic control group. This effect was most evident in the years immediately following the policy, as fossil sources temporarily replaced nuclear generation. However, over time, the gap between Germany and its synthetic counterpart narrowed, reflecting a gradual catch-up likely driven by the expansion of renewables. Our results suggest that the 2011 nuclear phase-out in Germany initially led to an increase in CO_2_ emissions from fossil fuels compared to a synthetic control group. This effect was most evident in the years immediately following the policy, as fossil sources temporarily replaced nuclear generation. However, over time, the gap between Germany and its synthetic counterpart narrowed, reflecting a gradual catch-up likely driven by the expansion of renewables. This convergence is consistent with the theoretical framework of carbon lock-in proposed by Unruh [2,3], who argues that energy systems are structurally locked into fossil technologies unless disrupted by strong exogenous shocks. In this context, the Fukushima disaster acted as such a shock, triggering Germany’s abrupt policy shift. This confirms the findings of Zhao et al. [4], who show that political sensibility for energy to be safe, accessible and environmentally not dangerous can intensify carbon lock-in.

The exit from lock-in in Germany was due to the Fukushima shock in 2011. The German case is interesting since after an initial sudden nuclear phase out, resulting in increased emissions, Germany was able to expand renewable energy generation and gradually reduce its reliance on fossil fuels, confirming the possibility of catching up in climate performance over the medium term.

A closer look at the emission components reveals that the initial increase in CO_2_ was mainly driven by coal, which peaked around 2013–2014. However, coal-related emissions declined sharply after 2016, and by 2023 they returned to levels either comparable or lower than the synthetic control. Oil-related emissions followed a similar trend, while gas emissions increased moderately, reflecting their role in ensuring grid stability during the transition.

Our analysis of the energy mix confirms that renewables played a dominant role in this catch-up, with a substantial and sustained increase in electricity generation from renewable sources between 2011 and 2019, surpassing the level of the synthetic control. Additionally, our findings show that CO_2_ emissions from gas slightly and continuously increased after the phase-out, following the growth in renewables, likely because gas was used to stabilize the grid during periods of low renewable output.

The key insight from this study is that the environmental cost of phasing out nuclear energy is not uniform over time: while the short-term costs in terms of CO₂ emissions are real and measurable, they can be absorbed in the long term through strategic investment in clean alternatives. Thus, if a nuclear phase-out is to be pursued, it should be carefully planned and accompanied by sufficient low-carbon capacity prior to the closure of nuclear plants. This approach minimizes the risk of fossil fuel lock-in and ensures alignment with climate goals.

## Supporting information

S1 Table Descriptive statistics. (DOCX)

S2 Table Synthetic Difference in Differences estimation results. Notes: Standard errors are calculated with the placebo procedures (12 repetitions), as suggested in Arkhangelsky et al. [28] for synth difference in differences with single treated unit. Standard error in parentheses. *** p < 0.01, ** p < 0.05, * p < 0.1.
(DOCX)

S1 Fig Per-capita electricity produced from nuclear power plants, kWh. (EPS)

S2 Fig Germany vs synthetic Germany, per capita carbon dioxide emissions from coal, oil and gas. Notes: The blue lines represent the per capita carbon dioxide emissions from coal, oil, and gas sources of Germany, while the the red line represents the per capita carbon dioxide emissions from each source of the synthetic Germany. The shaded area [27] is built by adding and subtracting to the outcome of the synthetic Germany one standard deviation of the difference between the outcome of Germany and synthetic Germany before the introduction of the policy.
(EPS)

S3 Fig Germany vs synthetic Germany, per capita electricity produced from coal, oil, gas and renewable energy sources, kWh. Notes: The blue lines represent the per capita electricity produced from coal, oil, gas, and renewable sources of Germany, while the the red line represents the per capita electricity produced from each source of the synthetic Germany. The shaded area [27] is built by adding and subtracting to the outcome of the synthetic Germany one standard deviation of the difference between the outcome of Germany and synthetic Germany before the i*ntroduction of the policy.*
(EPS)

S1 Data Dataset. Panel dataset used for the analysis. (CSV)
