# Supplementary material for: Nuclear phase-out: Can we catch up on CO2 emissions?
Source: PLoS One. 2025 Nov 10;20(11):e0336218. doi: 10.1371/journal.pone.0336218 (PMC12599922; doi:10.1371/journal.pone.0336218)
Supplement: S1 Table — (DOCX) [file pone.0336218.s001.docx]

| Variable | Obs. | Mean | Std. dev. |
| --- | --- | --- | --- |
| Per-capita CO₂ emissions from fossil fuel sources | 408 | 7.37 | 2.53 |
| Per-capita CO₂ emissions from coal | 408 | 2.85 | 2.03 |
| Per-capita CO₂ emissions from oil | 408 | 2.87 | 1.14 |
| Per-capita CO₂ emissions from gas | 408 | 1.65 | 1.15 |
| Per-capita electricity produced from fossil fuel sources (kWh) | 408 | 1944.08 | 1324.33 |
| Per-capita electricity produced from gas (kWh) | 408 | 785.20 | 929.41 |
| Per-capita electricity produced from coal (kWh) | 408 | 1634.11 | 1273.21 |
| Per-capita electricity produced from oil (kWh) | 408 | 309.97 | 316.19 |
| Per-capita electricity produced from renewable energy sources (kWh) | 408 | 1969.29 | 2459.35 |
| Per-capita electricity produced from nuclear power (kWh) | 408 | 2701.51 | 2162.22 |
| Gross domestic product per capita | 408 | 28470.05 | 15142.98 |
| Registered cars per capita | 408 | 0.62 | 0.15 |
| Per-capita total electricity consumption (kWh) | 408 | 7400.88 | 3837.02 |
| Average annual temperature (°C) | 300 | 9.18 | 3.15 |
| Share of industrial value added (% of GDP) | 384 | 26.47 | 5.33 |
| Population density (people per km²) | 408 | 132.22 | 120.72 |
| Share of population living in urban areas (%) | 408 | 71.08 | 10.92 |
| Share of electricity consumption by households (%) | 408 | 0.27 | 0.05 |
| Energy intensity (energy use per unit of GDP) | 276 | 4.44 | 1.31 |
