# Supplementary material for: Nuclear phase-out: Can we catch up on CO2 emissions?
Source: PLoS One. 2025 Nov 10;20(11):e0336218. doi: 10.1371/journal.pone.0336218 (PMC12599922; doi:10.1371/journal.pone.0336218)
Supplement: S2 Table — Notes: Standard errors are calculated with the placebo procedures (12 repetitions), as suggested in Arkhangelsky et al. [28] for synth difference in differences with single treated unit. Standard error in parentheses. *** p < 0.01, ** p < 0.05, * p < 0.1. (DOCX) [file pone.0336218.s002.docx]

|  | (1) | (2) | (3) | (4) | (5) | (6) | (7) | (8) |
| --- | --- | --- | --- | --- | --- | --- | --- | --- |
|  | Total per capita carbon dioxide emissions | | Per capita carbon dioxide emissions from gas | | Per capita carbon dioxide emissions from coal | | Per capita carbon dioxide emissions from oil | |
| End of sample: | 2023 | 2017 | 2023 | 2017 | 2023 | 2017 | 2023 | 2017 |
| Treated*post | 0.251 | 0.514** | 0.246 | 0.105 | -0.129 | 0.258 | 0.207 | 0.286** |
|  | (0.602) | (0.221) | (0.445) | (0.235) | (0.427) | (0.305) | (0.333) | (0.184) |
| Observations | 348 | 264 | 348 | 264 | 348 | 264 | 348 | 264 |
| Number of countries | 12 | 12 | 12 | 12 | 12 | 12 | 12 | 12 |
| Country and year FE | YES | YES | YES | YES | YES | YES | YES | YES |
